# Prevalence and Economic Burden of Osteoarthritis and Rheumatoid Arthritis in the Medically Underserved Rio Grande Valley: A Retrospective Longitudinal Analysis

**DOI:** 10.7759/cureus.74521

**Published:** 2024-11-26

**Authors:** John M Gaddis, Elias Arellano, Ryan Bialaszewski, Dominic Chau-Zanetti, Tyler Torres, Elizabeth Bergman, Kelsey Baker, Bharathi Gadad

**Affiliations:** 1 Orthopedic Surgery, The University of Texas Rio Grande Valley School of Medicine, Edinburg, USA; 2 Internal Medicine, The University of Texas Rio Grande Valley School of Medicine, Edinburg, USA; 3 School of Medicine, The University of Texas Rio Grande Valley, Edinburg, USA; 4 General Surgery, The University of Texas Rio Grande Valley School of Medicine, Edinburg, USA; 5 Physical Therapy, Texas Woman's University, Houston, USA; 6 Neuroscience, The University of Texas Rio Grande Valley, Edinburg, USA; 7 Psychiatry and Behavioral Sciences, The University of Texas Rio Grande Valley School of Medicine, Edinburg, USA

**Keywords:** ethnicity and arthritis prevalence, health disparities rio grande valley, medicare data analysis, osteoarthritis prevalence, rheumatoid arthritis cost

## Abstract

Introduction

Osteoarthritis and rheumatoid arthritis, two of the most common forms of arthritis worldwide, are chronic diseases affecting the joints. The Rio Grande Valley is characterized by an abundance of health disparities, with previous studies showing increased rates of multiple diseases and disorders in this region. This study aimed to determine both the prevalence and the risk-adjusted average cost of osteoarthritis and rheumatoid arthritis in the Rio Grande Valley and to compare them with the national average. We hypothesize that the prevalence and risk-adjusted average cost of osteoarthritis and rheumatoid arthritis in the Rio Grande Valley will be greater than the corresponding national averages.

Methods

Publicly accessible Medicare beneficiary data were utilized for our retrospective longitudinal, observational study. Osteoarthritis and rheumatoid arthritis data for risk-adjusted average total cost, overall prevalence, sex-stratified prevalence, and ethnicity-stratified prevalence, from 2012 to 2022, were compared between the Rio Grande Valley and the national means using specific domains of the “Mapping Medicare Disparities by Population” tool. Independent t-tests and a Mann-Whitney U test compared prevalence rates and risk-adjusted average total cost means, respectively, between the Rio Grande Valley and the national averages.

Results

Overall, the prevalence of osteoarthritis and rheumatoid arthritis in the Rio Grande Valley was significantly higher than the national average (39.9% vs. 26.9%, p < 0.001). Women in the region exhibited significantly higher rates of osteoarthritis and rheumatoid arthritis compared to the national average (47.6% vs. 32.1%, p < 0.001), and a similar trend was seen among Hispanic residents (41.6%) compared to the national mean (32.1%) (p < 0.001). Furthermore, the risk-adjusted average total cost for individuals residing in the Rio Grande Valley ($16,084.40) significantly exceeded the risk-adjusted average total cost nationally ($13,073.90) (p < 0.001).

Conclusion

In the Rio Grande Valley, there is an increased prevalence of osteoarthritis and rheumatoid arthritis compared to the national mean of Medicare beneficiary patients, particularly in women and those of Hispanic heritage. The substantial increase in risk-adjusted average total cost to treat osteoarthritis and rheumatoid arthritis highlights the economic burden faced by residents in the region.

## Introduction

Arthritis, a disease that affects the joints, can be categorized into two broad categories: degenerative arthritis and inflammatory arthritis [1]. Two of the most common manifestations of arthritis are osteoarthritis (OA), a form of degenerative arthritis, and rheumatoid arthritis (RA), a form of inflammatory arthritis [1-3]. OA is characterized by the chronic destruction of articular cartilage and is considered a disease of the entire joint [4-6]. The joints most commonly affected by this disorder include the knees, hips, hands, and spine [1,6]. OA can be divided into two subtypes: primary OA, which is idiopathic, and secondary OA, a sequela to other pathological processes such as trauma or previous disease [5,6]. Notable risk factors for developing OA include an increased age, obesity, female gender, genetic predisposition, and the biomechanics of the joint [5-7]. 

In contrast, RA is a systemic autoimmune disease that leads to an inflammatory response affecting synovial joints such as the hands, feet, and wrists while simultaneously damaging extra-articular organs [8]. Like many other autoimmune diseases, RA significantly affects females more than males [8,9]. RA is described as a multifactorial disease with an incompletely understood etiology, but genetic and environmental factors have been found to have a role in developing the disease [8,10]. For example, smoking is associated with an increased prevalence and severity of RA [8,10]. Both OA and RA have been found to cause significant morbidity and decreased quality of life [2-4].

Along the US-Mexican border, the Rio Grande Valley (RGV) spans four counties: Hidalgo, Cameron, Starr, and Willacy [11]. With a population that is 90% Hispanic, the RGV is the poorest region in Texas, with roughly 30% of the population living below the poverty line and 40% having no health insurance [11,12]. The RGV is a medically underserved area characterized by a shortage of healthcare professionals, centers, and services, with increased barriers to healthcare, including a lack of money, insurance, and transportation [11,13]. Furthermore, with the growing population of the RGV, primary care physicians (PCPs) are outnumbered, with a population-to-PCP ratio of 2,230:1, which makes access to rudimentary healthcare challenging [14]. Due to the lack of healthcare in the area, disorders such as osteoporosis, Kaposi sarcoma, type 2 diabetes mellitus, cervical cancer, and Alzheimer’s disease affect the population of the RGV at higher rates than the national average [11,12,15-17]. However, OA and RA (OA/RA) remain a less studied topic, with no prior studies exploring the prevalence and risk-adjusted average total cost (ATC) of OA/RA in the RGV. Therefore, we aimed to assess and compare the combined prevalence of OA/RA, along with the risk-adjusted ATC, in the four counties of the RGV with the national prevalence and risk-adjusted ATC of OA/RA from 2012 to 2022. This allows us to investigate further possible discrepancies in healthcare that affect the RGV. We hypothesize that the prevalence and risk-adjusted ATC of OA/RA among patients in the RGV will surpass that of the national average.

## Materials and methods

We performed a retrospective longitudinal analysis of the Medicare beneficiary unified dataset for OA/RA, spanning 2012 to 2022. For OA/RA data, we evaluated the prevalence and risk-adjusted ATC of the four counties within the RGV (Starr, Hidalgo, Willacy, and Cameron) compared to the national average. The impact of sex and ethnicity on prevalence was also evaluated. All collected data were de-identified, and the study was considered non-regulated human subjects research by the Institutional Review Board of the University of Texas Rio Grande University. 

We utilized the "Mapping Medicare Disparities by Population" tool on "Data.CMS.gov" to collect the data. The interactive tool required several specific parameters to access the particular dataset used for this study. The population was the Medicare Fee-For-Service from 2012 to 2022, with the measures being prevalence and risk-adjusted ATC. The Centers for Medicare and Medicaid Services (CMS) defines ATC as the yearly average of all costs related to a specific disease across all types of claims for beneficiaries [18]. Furthermore, the CMS determines risk-adjusted ATC as the product of risk scores generated at the beneficiary level by the CMS Hierarchical Condition Category risk adjustment model of specific subpopulations and the standard total cost of $9,276.26 [18]. We utilized the unsmoothed age-standardized adjustment, and the analysis included both the base measure and the difference from the national average. The domain comprised primary chronic conditions, the exact condition being OA/RA. The database did not provide a separate analysis for OA and/or RA alone; thus, we evaluated both conditions together in this study. Only Hispanic and White ethnicities were included in this study because the data for other ethnicities in the RGV were insufficient. Patients of all ages under the Medicare eligibility category were included in the study, and sex was categorized into three groups for each county: all, male, and female.

Data analysis was conducted using Anaconda Navigator version 2.5.0, Jupyter Notebook version 6.4.5, and Python version 3.9. Data frames were created with the respective prevalence data for the categories (all, male, female, White, and Hispanic) and the ATC (risk-adjusted). A Shapiro-Wilk test was utilized via the function scipy.stats.shapiro to ensure the data fit a normal distribution. Descriptive statistics for all variables were obtained via the function describe(). Additionally, the functions matplotlib.pyplot as plt, scipy.stats import ttest_ind, and scipy.stats.mannwhitneyu were used to create plots, run the independent t-tests, and conduct a Mann-Whitney U test, respectively, to obtain the data from the "Mapping Medicare Disparities by Population" tool. Independent t-tests were used to compare the prevalence means of the RGV and nationally for the categories (All, Male, Female, Caucasian, and Hispanic). A Mann-Whitney U test was used to compare the risk-adjusted ATC means between RGV and the national average. The a priori alpha level for significance was set at <0.05 for all statistical analyses.

## Results

Overall OA/RA prevalence

Table 1 presents the descriptive statistics for the prevalence and risk-adjusted ATC of OA/RA across the RGV counties and the USA from 2012 to 2022. 

**Table 1:** Descriptive statistics for OA and RA prevalence and cost by county in the RGV and USA. OA: osteoarthritis, RA: rheumatoid arthritis; SD: standard deviation; CI: confidence interval; RGV: Rio Grande Valley.

Variable	County	Mean (%)	SD	Lower CI	Upper CI
OA/RA overall prevalence rate	USA	32.09	2.21	30.61	33.58
	RGV	39.91	1.84	38.67	41.14
	Starr	48.64	2.29	47.10	50.18
	Hidalgo	37.27	2.57	35.54	39.00
	Cameron	36.45	1.37	35.54	37.37
	Willacy	37.27	2.28	35.74	38.81
OA/RA female prevalence rate	USA	32.09	2.21	30.61	33.58
	RGV	47.59	2.34	46.09	49.09
	Starr	57.36	2.73	55.53	59.20
	Hidalgo	44.82	2.60	43.07	46.57
	Cameron	43.82	1.60	42.74	44.89
	Willacy	44.36	2.98	42.36	46.36
OA/RA male prevalence rate	USA	32.09	2.21	30.61	33.58
	RGV	31.16	1.65	30.05	32.27
	Starr	38.55	2.66	36.76	40.33
	Hidalgo	28.82	2.48	27.15	30.49
	Cameron	27.64	1.21	26.83	28.45
	Willacy	29.64	1.69	28.50	30.77
OA/RA Hispanic prevalence rate	USA	32.09	2.21	30.61	33.58
	RGV	41.64	2.51	39.95	43.32
	Starr	49.27	2.69	47.47	51.08
	Hidalgo	39.09	3.53	36.72	41.47
	Cameron	38.45	1.69	37.32	39.59
	Willacy	39.73	3.20	37.58	41.87
OA/RA Caucasian prevalence rate	USA	32.09	2.21	30.61	33.58
	RGV	33.07	1.79	31.86	34.27
	Starr	36.36	3.59	33.95	38.77
	Hidalgo	33.55	2.02	32.19	34.90
	Cameron	32.00	1.41	31.05	32.95
	Willacy	30.36	3.26	28.17	32.56
Risk-adjusted ATC of OA/RA	USA	$13,073.91	$302.25	$12,870.86	$13,276.96
	RGV	$16,084.43	$1,005.05	$15,409.23	$16,759.63
	Starr	$16,159.82	$1,767.59	$14,972.33	$17,347.30
	Hidalgo	$15,710.36	$857.55	$15,134.25	$16,286.47
	Cameron	$15,077.91	$615.72	$14,664.26	$15,491.56
	Willacy	$17,389.64	$995.94	$16,720.56	$18,058.72

**Figure 1:**
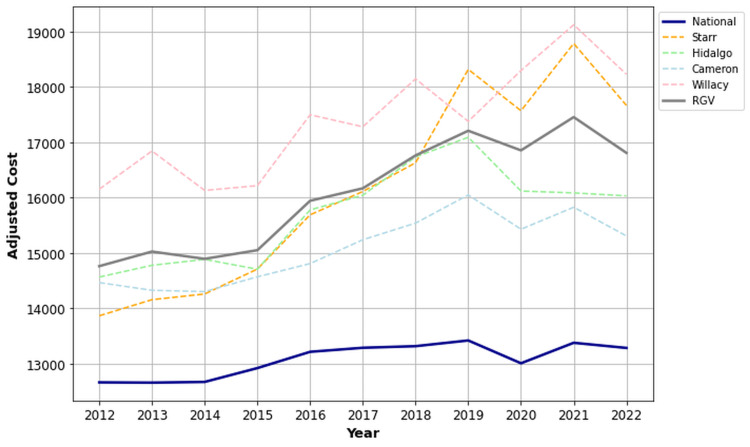
Prevalence of OA/RA in the RGV compared to the US national mean from 2012 to 2022. OA: osteoarthritis; RA: rheumatoid arthritis; RGV: Rio Grande Valley.

Residents of the RGV consistently experienced higher overall rates of OA/RA (39.9%) from 2012 to 2022, significantly surpassing the national mean (26.9%) (p < 0.001, 95% CI (38.7, 41.1)). Figure 1 illustrates the comparative prevalence rates of the RGV average, national mean, and individual counties. Across all counties, rates of OA/RA exceeded the national mean, with Starr County having the highest rates (48.6%). Interestingly, as shown in Figure 1, we found a dramatic decrease in the trend of overall OA/RA prevalence in the RGV, while the national mean steadily increased from 2012 to 2022. 

Sex-stratified OA/RA prevalence

**Figure 2:**
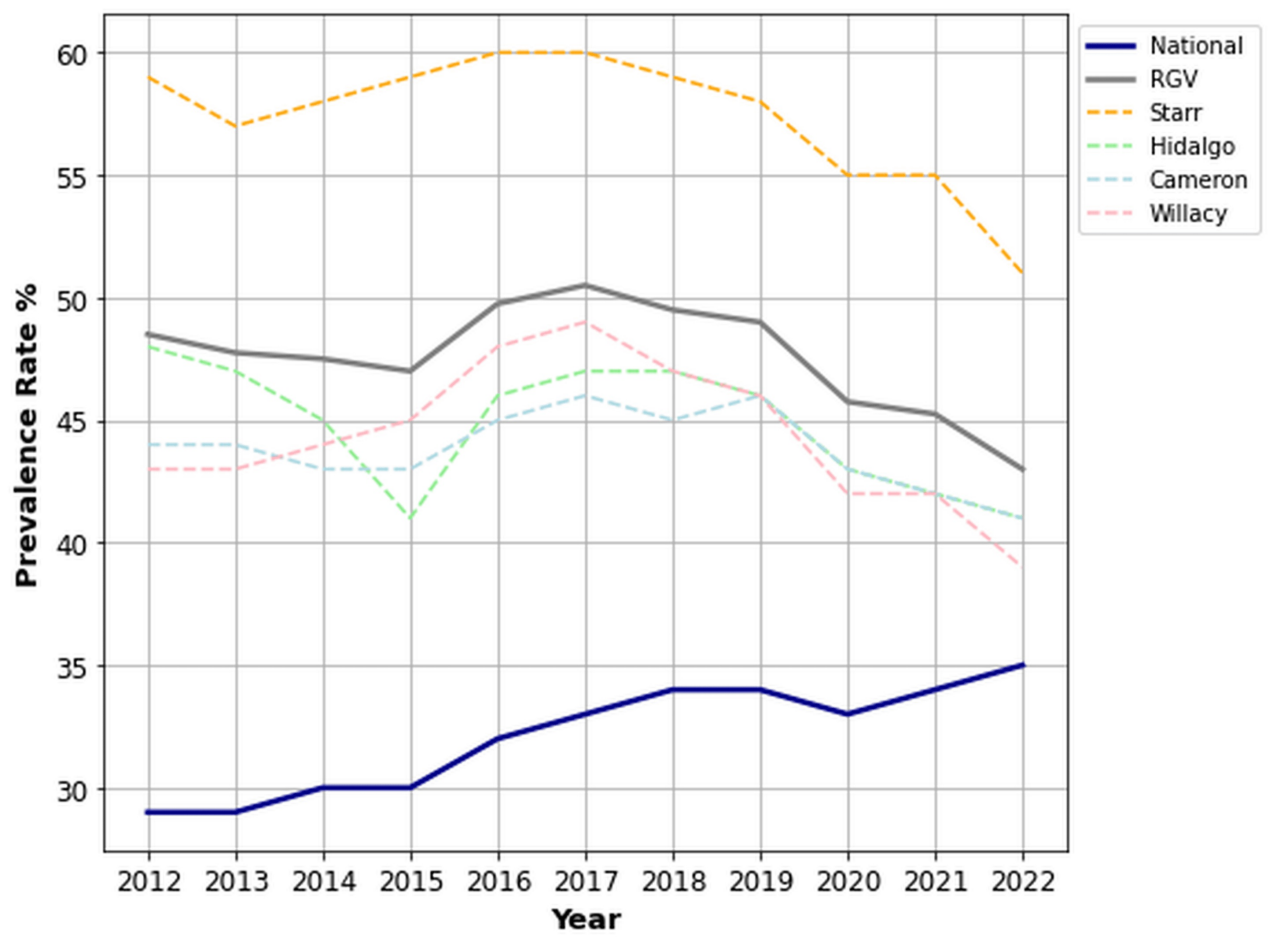
Prevalence rates of OA/RA for females in the RGV compared to the US national mean from 2012 to 2022. OA: osteoarthritis; RA: rheumatoid arthritis; RGV: Rio Grande Valley.

**Figure 3:**
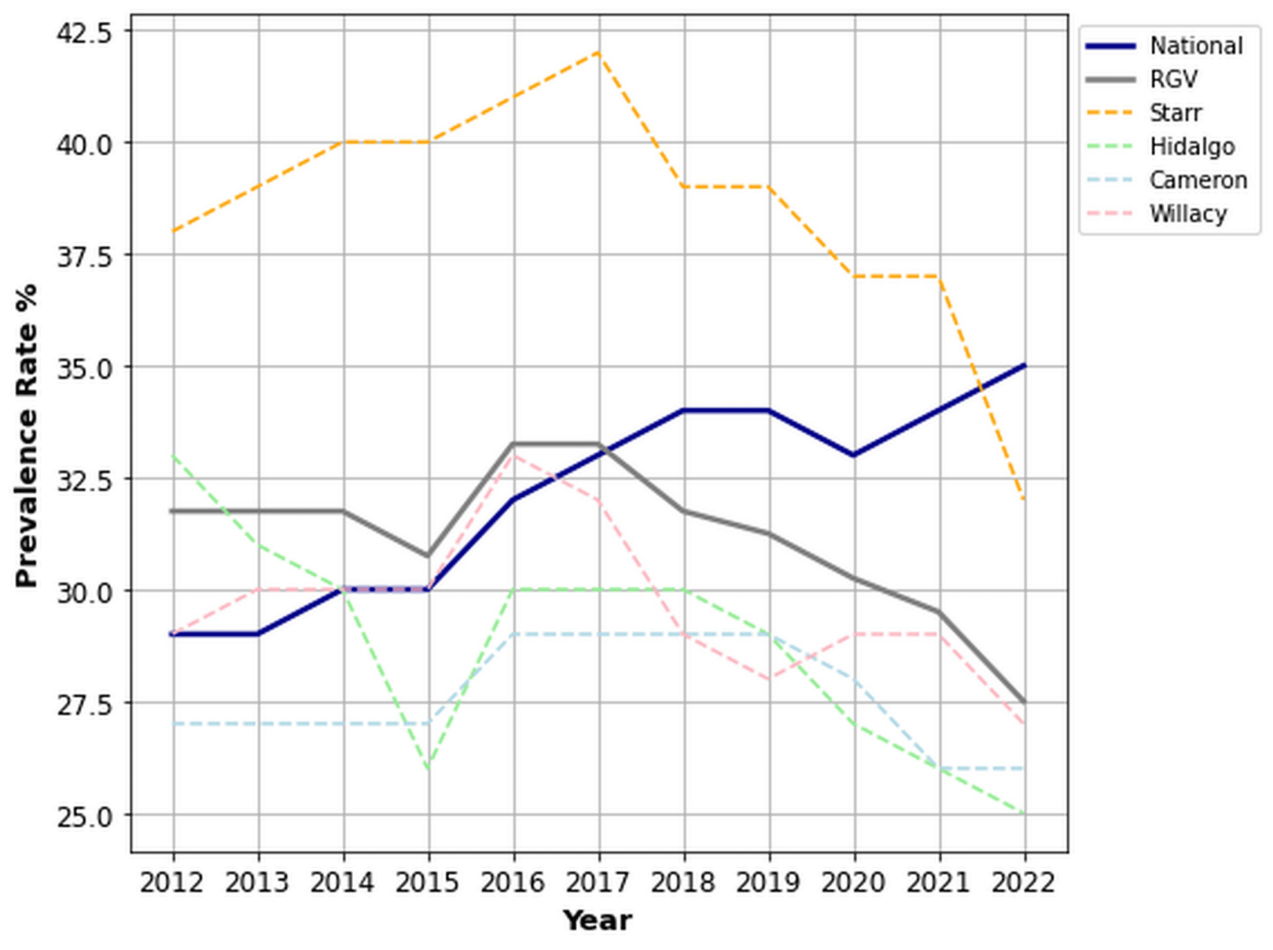
Prevalence rates of OA/RA for males in the RGV compared to the US national mean from 2012 to 2022. OA: osteoarthritis, RA: rheumatoid arthritis, RGV: Rio Grande Valley.

Females in the RGV demonstrated significantly higher rates of OA/RA than their national counterparts (47.6% vs. 32.1%, p < 0.001, 95% CI (46.1%, 49.1%)). Meanwhile, males in the RGV (31.2%) experienced no significant difference in the prevalence of OA/RA compared to the national mean (32.1%) (p = 0.276, 95% CI (30.0, 32.3)). Starr County reported the highest prevalence rates for both females (57.4%) and males (38.5%) within the RGV, and it was the only RGV county where male prevalence exceeded the national average (38.5% vs. 32.1%). Figures 2, 3 illustrate the female and male prevalence rates, respectively, across the RGV, national mean, and individual counties.

Ethnicity-stratified OA/RA prevalence

**Figure 4:**
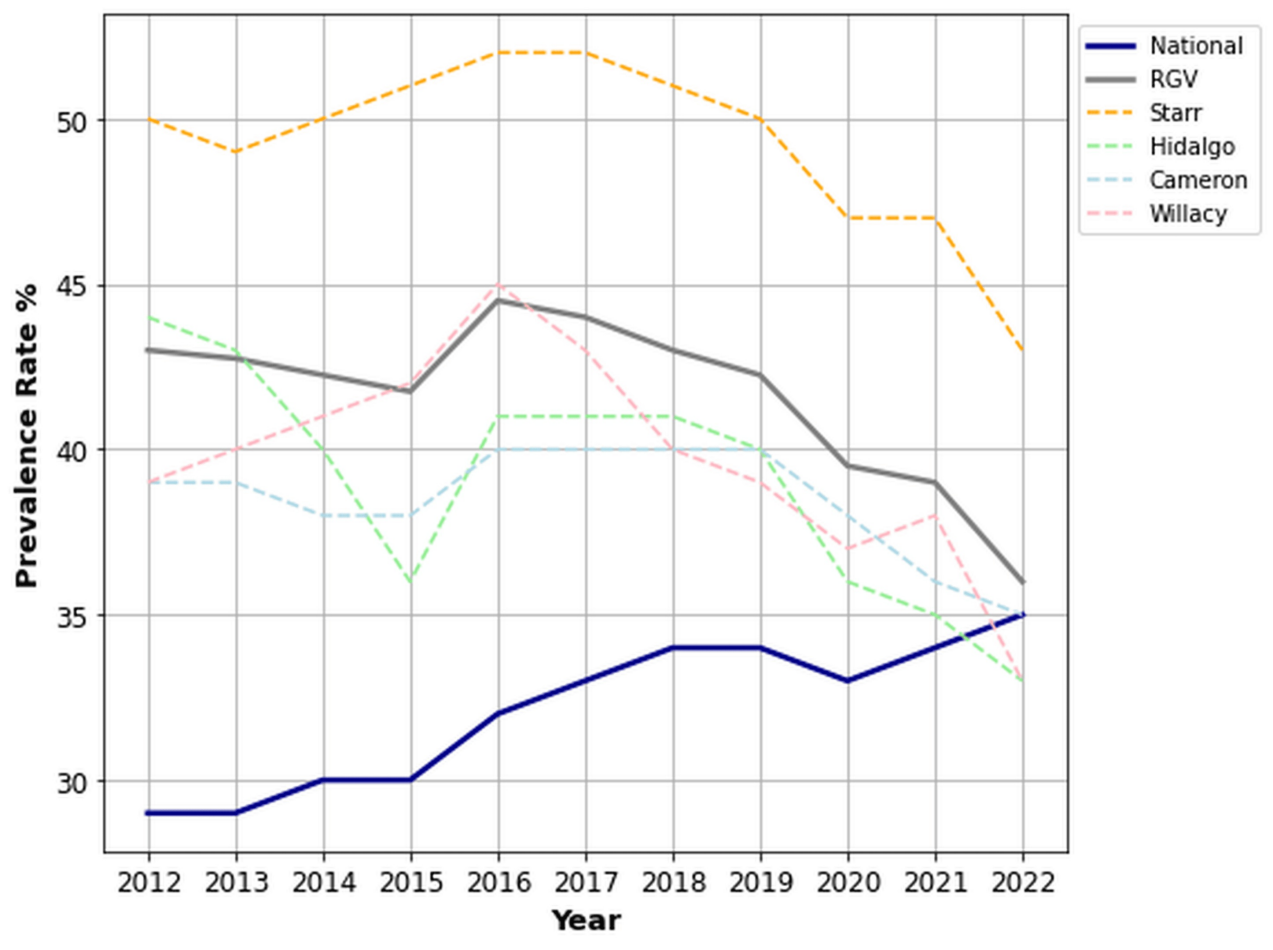
Prevalence rates of OA/RA for Hispanics in the RGV compared to the US national mean from 2012 to 2022. OA: osteoarthritis; RA: rheumatoid arthritis; RGV: Rio Grande Valley.

**Figure 5:**
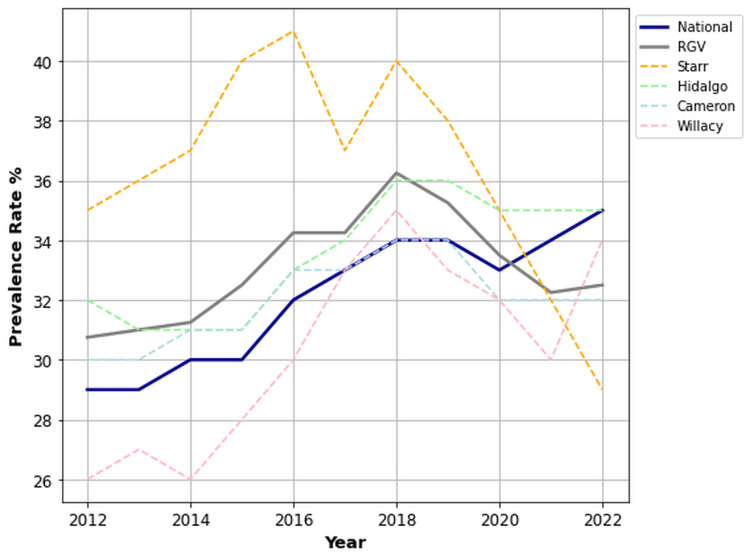
Prevalence rates of OA/RA for Caucasians in the RGV compared to the US national mean from 2012 to 2022. OA: osteoarthritis; RA: rheumatoid arthritis; RGV: Rio Grande Valley.

Figures 4, 5 illustrate the impact of ethnicity on prevalence rates of OA/RA across the RGV, national average, and individual counties. Hispanics in the RGV (41.6%) were affected by OA/RA at significantly higher rates than the national average (32.1%) (p < 0.001, 95% CI (40.0, 43.3)). When comparing ethnic groups within the RGV, Hispanics (41.6%) had elevated rates of OA/RA compared to Caucasians (33.1%). Caucasians in the RGV (33.1%) showed no significant difference in OA/RA rates when compared nationally (32.1%) (p = 0.268, 95% CI (31.9, 34.3)). Starr County had the highest prevalence of OA/RA among the individual counties of the RGV, with both Hispanics (49.3%) and Caucasians (36.4%) outpacing the national average (32.1%).

Risk-adjusted ATC of OA/RA

**Figure 6:**
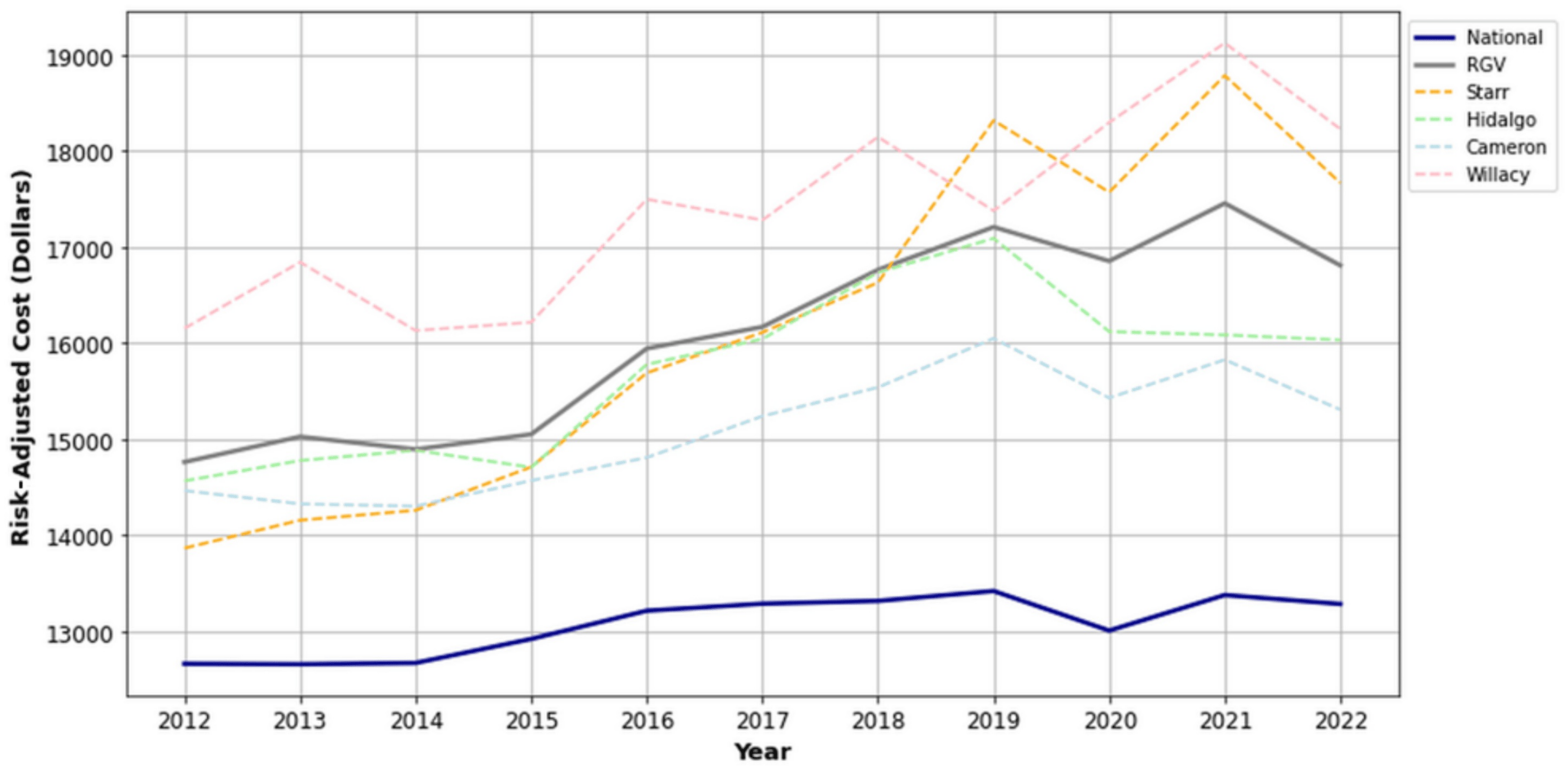
Risk-adjusted ATC of OA/RA in the RGV compared to the US national mean from 2012 to 2022. OA: osteoarthritis; RA: rheumatoid arthritis; ATC: average total cost; RGV: Rio Grande Valley.

The risk-adjusted ATC for residents of the RGV ($16,084.40) was significantly higher than the national risk-adjusted ATC ($13,073.90) (p < 0.001, 95% CI (15,409.20, 16,759.60)). Among the individual counties of the RGV, Cameron County had the lowest risk-adjusted ATC ($15,077.90) while Willacy County exhibited the highest risk-adjusted ATC ($17,389.60). All individual counties surpassed the national risk-adjusted ATC (Figure 6).

## Discussion

There are no recent reports on how the RGV is impacted by arthritis; however, this is a region with known healthcare disparities and low funding [17,19,20]. Therefore, we aimed to gain further knowledge regarding the prevalence and healthcare expenditures of OA/RA within the RGV compared to the broader national context. To our knowledge, our study is the first to report on the healthcare disparities surrounding OA/RA, specifically within the RGV. These data suggest that individuals residing in the RGV, particularly Hispanic females, are disproportionately impacted by OA/RA when compared to the rest of the nation. Furthermore, the healthcare expenditures for OA/RA are significantly higher than the national mean. 

OA/RA presents significant health concerns for the Hispanic population in South Texas, influenced by various factors such as low education levels, socioeconomic status, genetics, lifestyle choices, and cultural nuances. These challenges are particularly pronounced in the RGV, a region marked by widespread socioeconomic disadvantage and a large Mexican-American population, both of which contribute to heightened health risks [21]. In addition to grappling with a pronounced healthcare gap [19,20], the economic implications of OA/RA impose a noteworthy burden on RGV residents compared to the national average. Despite variations among individual counties within the RGV, all counties surpassed the national risk-adjusted ATC when treated for OA/RA. By highlighting these economic insights, we encourage healthcare providers to implement and further explore cost-effective interventions and targeted strategies to alleviate economic burdens within the RGV. Future research using individual-level population data could investigate whether residents in lower-income brackets face a higher risk of arthritis, providing valuable insights into the complex interplay of socioeconomic factors and arthritis in the region.

The observed decrease in the trend of overall OA/RA prevalence among residents in the RGV from 2012 to 2022 raises additional considerations regarding potential contributing factors as it directly contrasts with the national trend over the same period. One possible explanation could be through the development of modern medications, increased surgical interventions, and increased awareness initiatives, all of which could influence the decreased trend observed. An alternative and perhaps more plausible scenario, given the healthcare landscape of the region, is that the observed decrease may be due to reduced screening, limited access to care, lack of insurance, and fewer residents in the RGV seeking treatment for OA/RA [19,20]. In contrast, the downward trend could be attributed to positive initiatives implemented in the RGV, including the establishment of the University of Texas Rio Grande Valley School of Medicine, the presence of Area Health Education Centers located throughout the region, and UniMóvil, a mobile health clinic providing healthcare to the most underserved areas of the RGV [22-24]. However, given the healthcare barriers in the region and the persistently higher prevalence compared to the national average, continued scrutiny and targeted interventions are imperative for addressing OA/RA [25]. In this context, it becomes crucial to investigate and address potential issues related to screening and healthcare accessibility within the RGV, which may play a pivotal role in shaping the prevalence trends of OA/RA in the region going forward. Furthermore, the rising national mean prompts questions about the increase in OA/RA prevalence, whether it reflects a true surge in disease prevalence, enhanced diagnostic efforts, or other contributing factors requiring further investigation.

Current literature emphasizes the importance of the variability of OA and RA between different sex groups [26]. Sex-stratified analysis reveals consistent findings with females having a higher OA/RA likelihood; however, we identified a large disparity when examining the prevalence of OA/RA among females in the RGV (47.6%) compared to their national counterparts (32.1%). Furthermore, the distinct prevalence patterns observed in Starr County (57.4% for females) highlight the multifactorial contributions towards developing OA/RA and the need for further investigation into environmental factors influencing gender-specific disease manifestations. Moreover, the region is also impacted by increased OA/RA prevalence within specific ethnic groups, emphasizing the need for additional research in individual groups based on origin or background. Our findings indicated Hispanics in the RGV were disproportionately impacted by OA/RA when compared to the broader national context and even Caucasians in the same region. The elevated prevalence among RGV Hispanics emphasizes the necessity for tailored, ethnicity-specific healthcare considerations.

This study has inherent limitations. As a retrospective observational analysis relying on Medicare beneficiary data, the outcomes may not be universally applicable to all individuals in the RGV. The results could potentially misrepresent, or even underestimate, the prevalence of OA/RA, given the considerable portion of residents who lack legal status, face economic challenges, or lack healthcare coverage for adequate screening [20,27]. The study's inability to differentiate between OA and RA, stratify results for individual-level data, and factor in potential confounding risk elements due to database constraints poses a substantial challenge. Future research should consider integrating hospital-acquired data and community screening event information to enhance the breadth of findings, providing valuable insights into monitoring and treating individuals affected by OA/RA.

Furthermore, it is essential to emphasize that our study did not encompass all ethnic groups, as the "Mapping Medicare Disparities by Population" interactive tool lacked sufficient data for Black, Asian, and Native American residents in the RGV. Existing literature has highlighted an increased risk of OA/RA in African American and Native American populations, raising questions about whether this could exacerbate observed disparities within the region [28]. Given the predominant Hispanic composition of the RGV, constituting approximately 90% of the population, and considering the higher OA/RA rates among Hispanics, this demographic composition could potentially skew OA/RA rates.

## Conclusions

This study provides evidence for disparities in the prevalence and economic burden of OA/RA within the medically underserved RGV. Already faced with an affluence of healthcare disparities, the RGV also grapples with a significantly elevated burden of OA/RA, specifically among females and the Hispanic population. Despite a general decrease in OA/RA prevalence over the decade in the RGV, rates remain significantly higher than the national average. Conversely, the steady and significant increase in the risk-adjusted ATC in the RGV, when compared to the national average, accentuates the economic strain residents face. Our findings highlight the necessity of improved access to healthcare resources, the urgent need for public health initiatives, and the importance of interventions focused on mitigating financial constraints and improving health outcomes in a medically underserved community such as the RGV. 
